# A fast, machine learning‐based pipeline for atrophy subtyping and staging in Alzheimer’s disease

**DOI:** 10.1002/alz70861_108731

**Published:** 2025-12-23

**Authors:** Hannah Baumeister, Matthis Synofzik, Matthias Schmid, Oliver Peters, Julian Hellmann‐Regen, Josef Priller, Katharina Buerger, Robert Perneczky, Christoph Laske, Frederic Brosseron, Alfredo Ramirez, Annika Spottke, Jens Wiltfang, Anja Schneider, Stefan Teipel, Emrah Düzel, Frank Jessen, David Berron

**Affiliations:** ^1^ German Center for Neurodegenerative Diseases (DZNE), Magdeburg Germany; ^2^ Division of Translational Genomics of Neurodegenerative Diseases, Hertie Institute for Clinical Brain Research and Center of Neurology, University of Tübingen, Tübingen Germany; ^3^ German Center for Neurodegenerative Diseases (DZNE), Tübingen Germany; ^4^ Institute for Medical Biometry, Informatics and Epidemiology, University Hospital Bonn, Bonn Germany; ^5^ German Center for Neurodegenerative Diseases (DZNE), Bonn Germany; ^6^ Department of Psychiatry and Neurosciences, Charité Universitätsmedizin Berlin, Berlin Germany; ^7^ German Center for Neurodegenerative Diseases (DZNE), Berlin Germany; ^8^ ECRC Experimental and Clinical Research Center, Charité Universitätsmedizin Berlin, Berlin Germany; ^9^ Department of Psychiatry and Psychotherapy, School of Medicine and Health, Technical University of Munich, and German Center for Mental Health (DZPG), Munich Germany; ^10^ University of Edinburgh and UK DRI, Edinburgh UK; ^11^ Department of Psychiatry and Psychotherapy, Charité Universitätsmedizin Berlin, Berlin Germany; ^12^ Institute for Stroke and Dementia Research (ISD), University Hospital, LMU, Munich Germany; ^13^ German Center for Neurodegenerative Diseases (DZNE), Munich Germany; ^14^ Ageing Epidemiology (AGE) Research Unit, School of Public Health, Imperial College London, London UK; ^15^ Munich Cluster for Systems Neurology (SyNergy), Munich Germany; ^16^ Department of Psychiatry and Psychotherapy, University Hospital, LMU Munich, Munich Germany; ^17^ Section for Dementia Research, Hertie Institute for Clinical Brain Research and Department of Psychiatry and Psychotherapy, University of Tübingen, Tübingen Germany; ^18^ Department of Old Age Psychiatry and Cognitive Disorders, University Hospital Bonn and University of Bonn, Bonn Germany; ^19^ Excellence Cluster on Cellular Stress Responses in Aging‐Associated Diseases (CECAD), University of Cologne, Cologne Germany; ^20^ Department of Psychiatry and Glenn Biggs Institute for Alzheimer’s and Neurodegenerative Diseases, San Antonio, TX USA; ^21^ Division of Neurogenetics and Molecular Psychiatry, Department of Psychiatry and Psychotherapy, University of Cologne, Medical Faculty, Cologne Germany; ^22^ Department of Neurology, University of Bonn, Bonn Germany; ^23^ German Center for Neurodegenerative Diseases (DZNE), Göttingen Germany; ^24^ Department of Psychiatry and Psychotherapy, University of Göttingen, Göttingen Germany; ^25^ German Center for Neurodegenerative Diseases (DZNE), Rostock Germany; ^26^ Department of Psychosomatic Medicine, University Medicine Rostock, Rostock, Germany, Rostock Germany; ^27^ Institute of Cognitive Neurology and Dementia Research (IKND), Otto‐von‐Guericke University, Magdeburg Germany; ^28^ Department of Psychiatry, Medical Faculty, University of Cologne, Cologne Germany; ^29^ Center for Behavioral Brain Sciences (CBBS), Otto‐von‐Guericke University Magdeburg, Magdeburg, Sachsen Anhalt Germany; ^30^ Clinical Memory Research Unit, Department of Clinical Sciences, Lund University, Lund Sweden

## Abstract

**Background:**

Previous research has identified heterogeneous patterns of atrophy progression in individuals with Alzheimer’s disease, which are linked to interindividual differences in clinical presentation and cognitive decline. While such frameworks offer promise for individualized care and more targeted clinical trials, existing models usually require extensive pre‐processing and high‐field MRI. Here, we aim to improve accessibility of a previously proposed atrophy subtyping and staging model (Baumeister et al., 2024, *Brain*) by using accelerated pre‐processing and relying only on standard T1‐weighted images (1.5T or 3T).

**Method:**

We included participants with available T1‐weighted MRI scans from the DZNE Longitudinal Cognitive Impairment and Dementia Study (DELCODE; *N* = 813), the Alzheimer’s Disease Neuroimaging Initiative (ADNI; phases 1, GO, 2, and 3; *N* = 2,117), as well as the Anti‐Amyloid Treatment in Asymptomatic Alzheimer’s Disease and Longitudinal Evaluation of Amyloid Risk and Neurodegeneration (A4/LEARN) studies (*N* = 1,241). Images were acquired at 3T across all cohorts, except for *n* = 715 ADNI participants who were scanned at 1.5T. Atrophy markers were extracted using a deep learning‐based algorithm (FastSurfer; Henschel et al., 2020, *NeuroImage*), and the Subtype and Stage Inference (SuStaIn) algorithm (Young et al., 2018, *Nat Commun*) was used for atrophy progression modelling.

**Result:**

We replicated our previous finding of two distinct limbic‐predominant and hippocampal‐sparing atrophy progression subtypes in DELCODE using the updated model. At the individual level, subtype and stage classifications showed strong agreement with those from the original model. When retrained *de novo* in the independent validation cohorts (ADNI‐3T, ADNI‐1.5T, and A4/LEARN), the model identified comparable patterns of brain atrophy, highlighting its generalizability across diverse datasets and commonly used MRI field strengths. While subtype and stage assignments were based solely on cross‐sectional data, longitudinal MRI scans showed strong within‐subject stability of subtype classifications and monotonously increasing in atrophy stages, aligning with model assumptions.

**Conclusion:**

Assessing brain atrophy within the context of a global progression framework enables a more comprehensive evaluation than relying on single volume measures or isolated metrics. This work demonstrates that atrophy progression models can be adapted for real‐world settings, including those without access to high‐field MRI.

